# Understanding Migrant Farmworkers’ Health and Well-Being during the Global COVID-19 Pandemic in Canada: Toward a Transnational Conceptualization of Employment Strain

**DOI:** 10.3390/ijerph19148574

**Published:** 2022-07-14

**Authors:** Leah F. Vosko, Tanya Basok, Cynthia Spring, Guillermo Candiz, Glynis George

**Affiliations:** 1 Department of Politics, York University, Toronto, ON M3J 1P3, Canada; cjspring@yorku.ca; 2 Department of Sociology and Criminology, University of Windsor, Windsor, ON N9B 3P4, Canada; basok@uwindsor.ca (T.B.); ggeorge@uwindsor.ca (G.G.); 3 Études de la Pluralité Humaine, Université de l’Ontario Français, 9 Lower Jarvis St., Toronto, ON M5E 0C3, Canada; guillermo.candiz.1@ulaval.ca

**Keywords:** migrant farmworkers, Canada, COVID-19, employment strain, transnational lives, temporary labour migration, precarious status, work, health and well-being

## Abstract

During the COVID-19 pandemic, Canada imposed certain international travel bans and work-from-home orders, yet migrant farmworkers, declared essential to national food security, were exempt from such measures. In this context, farm worksites proved to be particularly prone to COVID-19 outbreaks. To apprehend this trend, we engaged an expanded and transnational employment strain framework that identified the employment demands and resources understood from a transnational perspective, as well as the immigration, labour, and public health policies and practices contributing to and/or buffering employment demands during and after the COVID-19 pandemic. We applied mixed methods to analyze administrative data, immigration, labour, and public health policy, as well as qualitative interviews with thirty migrant farmworkers employed in Ontario and Quebec. We concluded that the deleterious outcomes of the pandemic for this group were rooted in the deplorable pre-pandemic conditions they endured. Consequently, the band-aid solutions adopted by federal and provincial governments to address these conditions before and during the pandemic were limited in their efficacy because they failed to account for the transnational employment strains among precarious status workers labouring on temporary employer-tied work permits. Such findings underscore the need for transformative policies to better support health equity among migrant farmworkers in Canada.

## 1. Introduction

In Canada, the security of the local food supply and the survival of the agriculture industry rests on migrant workers. At the dawn of the COVID-19 global health pandemic, alongside introducing sweeping public health and safety restrictions, the federal government of Canada sought to manage the threats of national food shortages by supporting agricultural production and processing capacity in order to address an emerging backlog of produce through, most centrally, ensuring that growers maintained access to migrant farmworkers. At the same time, Canada’s federal labour department, Employment and Social Development Canada (ESDC), introduced policy measures aimed at protecting migrant farmworkers from risks associated with COVID-19, since they were documented to be prone to high levels of strain flowing from their precarious employment and residency status and particularly high risks of pre-pandemic injury and illness. Despite these efforts, while farms and greenhouses were declared essential worksites, they proved to be prone to COVID-19 outbreaks.

The majority of migrant farmworkers in Canada are legally authorized, working on employer-specific temporary work permits issued by the ESDC under the Temporary Foreign Worker Program (TFWP)—chiefly under its Seasonal Agricultural Worker Program (SAWP) and Agricultural Stream (AS). Such legal status can, in theory, offer opportunities for enhancing health equity within the temporary labour migration regimes; for instance, migrant farmworkers in Canada may be eligible for provincial public health insurance as well as injured workers’ compensation. However, their conditions of employment and residency can negatively impact their health and well-being on the job, off the job, and during periods of unemployment in myriad ways (e.g., [1,2,3,4]). As they labour transnationally, migrant workers’ lack of security of presence, often on account of the circularity of the labour migration programs in which they participate, can discourage them from voicing concerns or filing complaints about safety. Moreover, as we argue in this study, because these transnational workers originate predominantly in relatively low-income contexts, typically from Caribbean and Latin American countries affected by colonialism, they tend to tolerate working conditions that potentially compromise their health and well-being in order to maintain access to better paying Canadian jobs [5,6,7]. Furthermore, congregate employer-provided living accommodations, typical in agriculture, are often overcrowded and ill-equipped to provide for rest outside of work hours [8]. 

As we illustrate below, during the pandemic, when migrant farmworkers were deemed to be so essential that they were excluded from travel bans and border closures implemented to mitigate the spread of COVID-19, many of the conditions long-accepted as the status quo in farming continued to compromise workers’ health and well-being in the short, medium, and long term. Our analysis also boldly reveals how both longstanding and pandemic-specific immigration laws and policies, as well as public health mandates, which aimed to better protect migrant farmworkers from the risks to health and well-being associated with the job, not only failed but, in some cases, exacerbated the health inequities for this group.

To apprehend these trends and identify opportunities for better supporting migrant farmworkers’ health and well-being, in this study we engaged an expanded and transnational employment strain framework that identified the various physical, psychological, social, and organizational aspects of the work, as well as the immigration, labour, and public health policies and practices at the local, regional, national, and transnational levels, that contributed to and/or buffered employment strain—or the physical, social, and emotional costs of employment—during and after the COVID-19 pandemic. Employing this framework, we apply a mixed-methods analysis to administrative data, immigration, labour, and public health policy, as well as qualitative interviews with migrant farmworkers employed in Ontario and Quebec. Our central finding was that because the pre-pandemic conditions shaping migrant farmworkers’ employment and residency were at the root of the deleterious outcomes of the pandemic for this group, the effectiveness of the temporary band-aid solutions that failed to account for the transnational employment strains among the precarious status workers labouring on temporary employer-tied work permits and fixed-term employment contracts were limited and, in some cases, effectively heightened the employment strains and the risks to workers’ health and well-being that these strains produced. Such findings call for feasible yet transformative policy directions to better support the realization of health equity among migrant farmworkers in Canada. 

## 2. Materials and Methods

### 2.1. Design

This study utilized a dialectical, mixed-methods, team-based approach to explore the relationship between the structure and operation of Canada’s TFWP, the migrant farmworkers’ risks of contracting COVID-19, and their experiences working and living in Ontario and Quebec during the pandemic. Adopting an anti-colonial, transnational, and anti-racist standpoint, the insights we found flow from the “active mixing” of critical public policy analysis with insights from migrant farmworkers, a recursive and dialogical strategy that aims to yield practical alternatives directed at transformative change [9,10]. In conducting our research, we relied on multiple modes of inquiry: a statistical analysis of administrative data, drawing on customized data requests from Immigration, Refugees and Citizenship Canada (IRCC) and publicly accessible data; a policy analysis, examining the federal and provincial laws and policies directed at migrant farmworkers issued principally in response to the COVID-19 crisis and submissions to and minutes of the board meetings of local public health units; and semi-structured interviews conducted between October 2021 and February 2022 with thirty migrant farmworkers enrolled in two streams of Canada’s TFWP, as well as non-status migrants employed in agriculture in Quebec and Ontario, Canada’s two most populous provinces and those in which a majority of migrant farmworkers in Canada are employed. In 2020, for instance, 50,126 temporary migrant farmworkers were employed in Canadian agriculture. That year, approximately 22,834 of such workers (or 45.6% of all migrant farmworkers in Canada) worked in Ontario, while 13,094 (or 26% of all migrant farmworkers in Canada) worked in Quebec [11]. In the province of Quebec, we conducted the interviews in the Capitale-Nationale region and in the Montréal region. In the case of Ontario, we conducted all but one interview in the Leamington area and one interview in the Niagara region. This interview component was approved by the University of Windsor Research Ethics Board REB# 21-174.

### 2.2. Sampling

In conducting open-ended in-depth interviews, we sought to capture workers’ perceptions and experiences of their work and residency and to explore the nature of the social phenomena under study, instead of setting out to “test” hypotheses about them [12] (p. 248). In so doing, we employed purposeful sampling [13]; to recruit participants, we relied on several strategies, including our previous contacts among migrant workers as well as referrals by migrant support organizations in Ontario and Quebec. In Quebec, RATTMAQ (Assistance Networks for Migrant Agricultural Workers in Quebec) played a vital part in our research. Not only did RATTMAQ put us in touch with many migrants, but a staff member from this organization also emphasized the importance of our research project to workers, thus encouraging them to share their stories with us. To recruit other participants, we used snowball sampling.

### 2.3. Data Collection

To ensure the comfort of interviewees and protect their anonymity (all names utilized in this article are pseudonyms), the interviews were carried out in different places, for example, in public spaces, in the homes of workers, on the premises of support organizations, and in the workplace. We also conducted some interviews remotely through the WhatsApp application. All interviews were digitally recorded with interviewees’ consent.

### 2.4. Sample Description

Among the thirty workers interviewed in this study, thirteen were employed in Quebec at the time of the study, and seventeen were employed in Ontario. With respect to the country of origin, our sample was diverse: eight workers were from Mexico, fifteen were from Guatemala, two were from Honduras, four were from Jamaica, and one was from the Philippines. Most of the interviewed migrant farmworkers were employed on a TFWP contract, either under the SAWP or the AS. Notably, the migrant farmworkers from Guatemala were overrepresented in the sample because Guatemalan farmworkers, admitted under Canada’s AS, predominate in Quebec. In 2019, of the total number of 16,525 migrant farmworkers participating in Canada’s Temporary Foreign Workers Program in Quebec, 58% (a total of 9620) were from Guatemala, all of whom were recruited under the AS. Meanwhile, Mexican workers employed on the SAWP made up 36.5% (a total of 6025) of all temporary workers in Quebec [14]. Reflecting the source country representation in our sample, the workers recruited under the AS constituted the majority of all interviewees. Only seven migrant workers interviewed in our study had been recruited under SAWP. To gain a greater understanding of the experience of undocumented migrants during the COVID-19 pandemic, we also interviewed one migrant farmworker who was not on a TFWP contract.

Most of the thirty workers in our sample self-identified as men, which was as expected given that this labour force is predominantly male. For instance, only 7.6% of foreign workers in agriculture were women in 2017 [15]. Almost half of the workers (16 out of 30) were married, and the rest were single, divorced, or separated. Their ages ranged between 21 and 65 years old, with 36 being the average age. Seven participants had primary, eight had secondary, and fourteen had post-secondary education. Five had no children, but among those who did have children, the number of children ranged between one and four. The length of work in Canada ranged between one year and thirty-one years. The three workers who had worked in Canada the longest had been employed on Canadian farms for 31, 27, and 21 years individually.

### 2.5. Analysis

The interviews were coded using a qualitative coding frame [16] and analyzed using thematic analysis [17], a flexible, recursive strategy that lends itself to the dialogical and dynamic approach we adopted in this study while also allowing us to foreground the way the workers narrated their experiences of work and COVID-19 to render the visible linkages, incongruities, perceptions, and silences between policies and practices and the lived experiences of migrant farmworkers. While the guide structuring our interviews derived insight from the employment strain model, the interviewing strategy we undertook sought to activate narratives that included the unique dimensions of migrant farm work (such as employer-provided housing) that transcended the employment strain model. The open-ended nature of this strategy also enabled us to reveal the need to expand the dominant notions of the employment strain to reflect the experiences of interview subjects (i.e., migrant farmworkers).

## 3. Toward a Theory of Transnational Employment Strain: Centering Migrant Farmworkers Navigating Precarious Jobs and Residency Status

**Figure 1:**
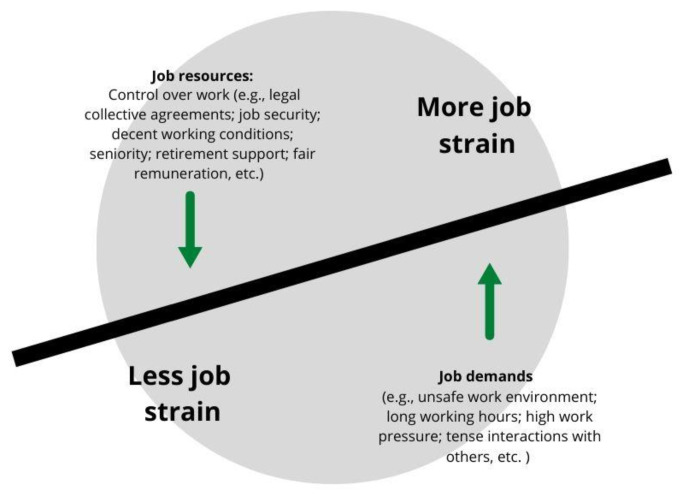
Job strain among (national) citizen-workers engaged in full-time permanent ongoing employment.

As a framework for analyzing the relationship between health and work among migrant farmworkers in Canada during the COVID-19 pandemic, we take up yet depart from the notion of *job strain* that is prominent in the literature on work and health—that is, the balance (or lack thereof) between job demands and job resources [18,19]. Job demands, understood as “physical, psychological, social, or organizational aspects of the job that require sustained physical and/or psychological (cognitive and emotional) effort or skills”, are present in all occupations and can be linked to certain physiological and psychological costs that contribute to job strain [18] (p. 312). In turn, job resources, such as fair remuneration, job security, and opportunities for promotion can serve to buffer the impact of job demands’ contributing to job strain [19,20] (Figure 1). The job strain model is useful for understanding how paid work intertwines with health and well-being, providing a framework for understanding how the balance between resources and demands can further and/or limit inequities in health.

**Figure 2:**
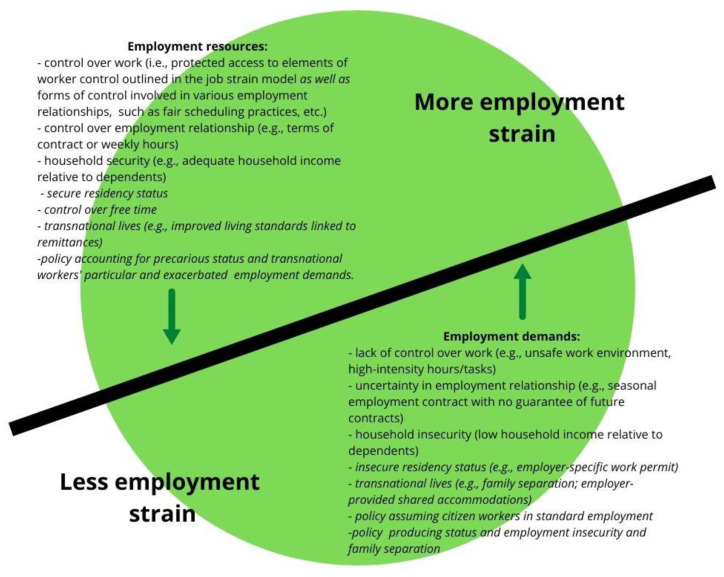
Transnational employment strain among workers both with various residency statuses and engaged in diverse employment relationships.

However, because this model assumes a (national) citizen-worker holding a full-time, continuous (i.e., permanent) job complete with a suite of entitlements as well as assumed supports associated with the heteronormative nuclear family form [21], it fails to account for the key factors contributing to strain. For this reason, we took up an expanded notion of “employment strain” devised and advanced in scholarship on precarious employment [22,23], which extends the job strain model to include employment *relationships* (i.e., to look beyond a full-time continuous job with a single employer at a fixed worksite), household insecurity, and employment supports, while attending to axes of differentiation linked to social location, and migration/citizenship status in particular [23]. For migrant farmworkers, employed on fixed-term employer-specific contracts, their *precarious residency status* is a constitutive feature of the employment strain they experience. Furthermore, unlike the job strain perspective, the employment strain model also accounts for the demands and resources often viewed to be outside the realm of paid work (i.e., within the realm of the household) and the demands and resources related to the relations of distribution [23], such as household earnings, extended health benefits, and dependent household members [22]. As we contend in this article, an analysis of migrant farmworkers’ demands and resources arising from household relationships also needs to account for these migrants’ *transnational lives* and *relationships arising from the circularity of this migratory movement*. This expansive framework led us to employ a broader understanding of the demands and resources that we label *employment demands* and *employment resources*. Finally, extending the employment strain framework to account for the structural context, we suggested that this perspective ought to include policies that either reduce or amplify employment strains (Figure 2). Thus, to evaluate the forces shaping migrant farmworkers’ health and well-being, we attended to the intersection of their precarious residency status and transnational lives, alongside the particular policies that govern their work and residency.

Within a policy context that prioritizes national sovereignty, migrant farmworkers’ fixed-term employer-specific employment contracts foster an ever-present threat of repatriation to one’s country of origin, otherwise known as “deportability” [24,25,26], heightening the levels of employer control [27] and impacting the workers’ well-being in a multitude of ways. The fear of an employer’s reprisal prompting repatriation and/or exclusion from future seasonal employment contracts inhibits migrant farmworkers from filing complaints or calling on employers to address safety issues, for example (e.g., [24,25,26]). Threats and acts of deportability can also place pressure on migrant farmworkers to conceal an injury or illness out of fear of medical repatriation or exclusion from future employment contracts on account of presumed injuries or poor health [28,29]. Language barriers and lack of access to language training on account of insecure residency status can circumvent migrant farmworkers’ ability to voice concerns about workplace safety and otherwise establish clear lines of communication with managers and employers [30]. Moreover, migrant farmworkers’ conditions of residency and employment relationships can also distort the intended effects of certain employment resources designed for citizens and permanent resident workers. For instance, although legally authorized migrant farmworkers pay into Canada’s Employment Insurance program, an income support program available to workers in case of illness, unemployment, or parental leave, their ability to access this program is compromised by their seasonal- and contract-based employment contracts and precarious residency status. As a result, migrant farmworkers’ inclusion in this program only serves to exacerbate their employment strain insofar as paying premiums further squeezes their already relatively low wages without any future opportunities for recuperating their losses through income benefits [31]. 

For migrant farmworkers who come to Canada on temporary work permits and leave their families and communities behind, employment demands and resources do not take shape exclusively at the national level but rather need to be understood within their transnational context. Within the Canadian immigration policy context, family accompaniment for TFWP-participants is not permitted, and, as a result, migrant farmworkers maintain economic and social relationships with their household members across transnational space. These transnational relationships can be both a source of increased employment demand (e.g., the emotional toll of separation and lack of social support) and an employment resource (e.g., improved living standards linked to remittances). The ways in which transnational relationships function as both sources of increased employment demand and also employment resources among migrant farmworkers is akin to how a previous study [32], in a recent issue of this journal, critiqued the applicability of the job strain model for home care aides, underlining its failure to account for the intrinsic rewards and pride in care work and effectively advocating the adoption of a more relational approach to conceptualizing the rewards of caring labour vis-à-vis job stress). The reliance on remittances may also impact the way migrant farmworkers respond to other employment demands. Furthermore, without their household members present in Canada, the migrant farmworkers are placed in employer-provided housing they share with their co-workers, with whom they may have no prior relationships. Their housing conditions as well as their relationships with their co-workers, therefore, represent additional employment demands that impact the migrant workers’ health and well-being, as discussed in more detail below.

During the COVID-19 pandemic, these and other longstanding demands persisted and, in some cases, intensified, despite government efforts to better protect these essential migrant workers from transmission and infection of the virus. In particular, the pandemic increased employment strains in four ways: it introduced the new risk of COVID-19 transmission in workplaces and in employer-provided dwellings; it magnified the mental and physical health risks associated with employer-provided housing; it heightened the fears of employer reprisal and repatriation; and it reduced earnings and/or introduced new threats of lost income. Accordingly, our engagement of a transnational employment strain perspective sought to address how longstanding immigration and labour laws, policies, and practices, persisting alongside COVID-19-specific public policy interventions directed at improving the quality of and access to resources for migrant farmworkers, contributed to magnifying such strains among this group of transnational workers. To evaluate whether and the degree to which employment resources, both longstanding and pandemic specific, buffer these demands, it was also necessary to center an insecure residency status and the transnational nature of migrant farmworkers’ lives. In addressing these components of employment strain among this group of workers and attending to the demands, resources, and strains in a transnational context, rather than exclusively national, the limits of prevailing resources in buffering their effects are rendered more visible and complex.

## 4. Migrant Farmworkers in Canada: Conditions of Entry, Stay, and Work

Migrant farmworkers in Canada include both legally authorized migrants entering under temporary labour migration programs designed to manage migration and migrants labouring without a valid work permit. While the data on workers without legal status in Canadian agriculture are limited [33], there are thousands of migrants working without work permits across the country; some estimates claim that up to 2000 “undocumented” workers are located in the Ontario farming region of Windsor-Essex alone [34], a central location in which we conducted semi-structured interviews. However, a majority of the over 60,000 migrant farmworkers in Canadian agriculture are legally authorized. This group entered Canada principally through two TFWP subprograms—the SAWP and the AS. In 2021, 34,270 migrant farmworkers entered Canada under the SAWP, whereas 26,730 entered under the AS [35]. 

Canada’s largest and most longstanding program under the TFWP, the SAWP, has operated to meet agricultural employers’ need for low-wage, flexible labour on a seasonal basis without interruption since 1966. It enables circular migration and functions through agreements between the governments of Canada and Mexico and Canada and Caribbean states; Mexico and Jamaica are the predominant countries of origin for SAWP participants [35]. Bilateral agreements set out the terms and conditions under which the migrant farmworkers drawn from participating countries migrate to Canada temporarily.

Temporary work permits provided under the SAWP allow for a maximum 8-month stay. They are also employer-tied and permit growers to terminate and effectively repatriate workers prior to the expiration of their work permits if insufficient work is available or for other reasons (e.g., illness or injury) [5,24,26,27]. At the same time, the SAWP permits circularity or rotation; that is, SAWP participants that do not confront these obstacles are enabled to return year after year and many, including many in our sample, take part in the program long-term. Quite uniquely, unlike other many other international mobility and temporary migrant work programs, the SAWP does not allow for spousal or family accompaniment, even though its recruitment policies prioritize workers with dependents [36]; paradoxically, as some scholars have noted, this recruitment strategy works to ensure participants’ annual return to countries of origin [37,38,39]. 

In addition to the SAWP, Canadian growers may recruit migrant labour through the AS. Unlike the SAWP, bilateral agreements between Canada and sending countries do not underpin the AS, and Guatemala is the top source country for this program (although the participation of workers from Mexico, India, and Jamaica rose substantially in the late 2010s) [35]. The AS provides work permits for a maximum of 24 months (also with no option for spousal or family accompaniment). Moreover, the work permits issued to migrant workers under the AS are akin to those of the SAWP in that they are tied to specific employers. Thus, while they reside in Canada, under both the SAWP and the AS, migrant farmworkers are not permitted to circulate freely in the labour force and even face constraints in transferring between agricultural employers (see for e.g., [40] (pp. XV 1–3). 

## 5. Results and Discussion

### 5.1. Transnational Employment Strain Pre-Pandemic

Pre-pandemic, as non-citizens authorized to work on fixed-term employer-specific contracts separated from their household and communities across transnational space and placed in employer-provided congregate housing, migrant farmworkers faced many demands and limited resources to buffer them. Chief among the employment demands were unsafe working environments, long work hours, workplace pressure, and substandard living conditions in employer-provided housing. Agricultural work is one of the most dangerous occupations in Canada [41]. Occupational hazards may include exposure to toxins, extreme temperatures, and adverse climatic conditions, and musculoskeletal injuries [42]. In fact, Quebec’s Administrative Labour Tribunal (TAT) recognized non-Hodgkin’s lymphoma as an occupational injury caused by exposure to pesticides [43]. It is therefore not surprising that more than half of the workers we interviewed identified occupational risks in their workplaces. Among them, the exposure to dangerous chemicals (i.e., pesticides) was mentioned most frequently, mainly by the Ontario workers who were employed in greenhouses. However, it was not the use of the pesticides but rather the violation of safety procedures that was of major concern to the workers. For instance, the workers reported having to be present while greenhouses were sprayed. Other risks involved extreme climatic conditions and muscular injuries. At the same time, when the workers experienced minor injuries, they often did not receive medical care. These findings are consistent with previous research illustrating the health risks that emerge due to the migrant farmworkers’ heightened dependence on employers to access healthcare [3,44]. Furthermore, workplace harassment, reported by some of the migrant workers we interviewed, as well as other researchers (e.g., [27]), makes the unsafe work environments particularly toxic. At the same time, the provincial legislation concerning occupational health and safety fails to adequately protect the farmworkers [45,46,47], and indeed, research shows that many agricultural worksites are not in compliance [48]. 

Long working hours is yet another demand that we and other researchers (e.g., [4,27,49]) found to be widespread among the migrant farmworkers in Canada. Most of the workers in our study told us that they worked between 50 and 60 h per week, often without a full day of rest. Other studies (e.g., [50]) similarly reported that on average, the respondents worked 64.2 h per week, and in peak harvest seasons, 90% of them worked even longer hours, between 61 and 80 h a week. At the same time, many of the workers we interviewed told us that they were expected to work at a fast pace and keep their breaks to a minimum, at times, even shorter than those required by law [24]. 

Still, none of the possible *employment-specific resources*, such as control over one’s work environment, participation in decision-making, fair renumeration, job security, opportunity for promotion, or co-worker support were available to the workers we interviewed in this study. In fact, not only did the workers receive low pay, but their jobs were contingent upon the arbitrary power of the employers to keep them on a contract for which the work visas were granted, a fundamental feature of their unique experience of *employment strain* [22,23]. That is, the profound insecurity of the workers’ current and future employment in Canada, in the context of immigration policies that grant migrant farmworkers no more than a temporary status contingent upon their satisfactory performance, conditions migrant farmworkers’ responses to the demands. For instance, the fear of being dismissed by their employers and returned to their home countries prevents some workers from reporting workplace injuries, ailments, or unsafe working or living conditions (e.g., [3]). Reflecting how employment relationships and insecure residency status function as interlinked demands, most migrant farmworkers also comply with requirements to work faster and with a dearth of breaks to avoid being dismissed and deported [24,25,26]. These conditions, which compel workers to become hyper-productive [8,37], thus intensify the already excessive employment demands. Most migrant workers interviewed in our study were aware of the fact that their continued participation in the program was contingent upon their ability to work well and fast, and they did not wish to jeopardize their current or future employment in Canada. Migrant farmworkers’ fear of reprisals also makes largely ineffective such policy interventions as the open work visa for vulnerable workers who are victims of abuse, introduced by the government of Canada in 2019 [51], since very few workers are willing to risk their status in the program by complaining against their employers. For the same reason, complaint-driven workplace inspections, the backbone of the occupational health and safety and employment standards enforcement system, fail to protect workers with precarious employment and legal status since they are unlikely to use these mechanisms to complain about unsafe working conditions [52]. In other words, policy interventions that are meant to be an employment resource for the farmworkers have largely failed to buffer against their employment demands precisely because they have not accounted for the fundamental feature that constitutes their employment strain: their precarious employment and legal status.

As an illustration of the need to center migrant workers’ transnational lives and relationships while analyzing the link between health and work, for most of the workers we interviewed, the main reward of working far away from their home is the opportunity to improve their families’ standards of living and provide education to their children [38]. Thus, given the wide gap in income earning opportunities and wages between Canada and migrants’ home countries, the transnational financial support that migrants provide to their families is an “employment resource,” albeit one shaped by global inequalities. In fact, most of the workers in our study told us that they preferred to work long hours to maximize their remittances. On the other hand, the strong commitment to their families undermined the workers’ ability to resist excessive employment demands. Workers “perform submission” to their employers [37] and rarely speak out against unsafe conditions or abuse they face. Furthermore, the emotional cost of family separation across transnational space [38] can be seen as an additional job strain that undermines migrant farmworkers’ well-being; being separated from their household members and communities across transnational space deprives migrant workers of another possible buffer against employment demands, namely, essential resources that these support networks might provide for their everyday and long-term renewal as workers.

All of the migrant farmworkers we spoke to reported living in congregate housing. As this housing is typically located on a farm, work and home are merged “into one geographic site” [8]; as such, the aforementioned housing conditions constitute an additional employment demand. Similar to the experiences of migrant farmworkers chronicled in other studies (e.g., [53,54]), many migrant farmworkers we interviewed complained about overcrowded and dilapidated housing, lacking in essential resources such as hot running water, kitchen supplies, proper ventilation, adequate toilets and showers, and laundry that allowed for no privacy and where conflicts between migrant workers, forced to share scarce facilities and amenities, compromised workers’ mental strain [8]. Although employers are required to have housing inspected annually, the poor quality inspections and inadequate resources for and practices surrounding enforcement allow for the sustainment of these health-compromising and interpersonally taxing conditions that contribute to employment strain [55]. Furthermore, as they were forced to share a living space with co-workers with whom most had no prior relationships, the migrant farmworkers in our study reported tensions and hostilities arising from the competition for the use of appliances and amenities.

Despite these conditions, the migrant farmworkers reported that few meaningful public policy interventions were implemented to mitigate their effects. The Canadian immigration policies that tie the temporary residency status of migrant farmworkers participating in the SAWP and the AS to their employers, who thereby have the unilateral power to keep them in the program or deport them [24,25,26], separate the workers from their families [38] and thereby externalize their social reproduction—or their ability to maintain themselves and their families materially and psychically on both daily and intergenerational bases—to their home communities [31]. These policies, which also deprive SAWP and AS participants of the opportunity to settle permanently in Canada [56], are largely responsible for the ways in which migrant farmworkers experience transnational employment strains. While the Canadian government put in place certain measures to mitigate these employment strains, such as the 2015 amendments to the 2002 *Immigration and Refugee Protection Act (2002)* that introduced minimalist regulations to reduce exploitation and enforce workers’ rights for workers employed under the TFWP and the Open Work Permit for Vulnerable Workers program, introduced in 2019, that aims to provide open work permits to workers deemed to be “experiencing or at risk of abuse” [51], these regulations are insufficient at best, as they are “flawed by design” [57,58]. Despite their aims, such government-provided resources overall fail to challenge or account for the migrant farmworkers’ insecure residency status and transnational lives—the key factors shaping the employment strain they confront.

The COVID-19 pandemic amplified employment strain amongst workers by making their working and living environments even more perilous due to the potential for the virus to spread in workplaces and in employer-provided dwellings. However, the federal, provincial, or regional authorities were ineffective in securing migrant farmworkers’ safety. In Ontario alone, it is estimated that over 1000 migrant farmworkers tested positive for COVID-19 between April and July 2020 [59,60]. Provincially, the rate of infection among the general population was approximately 250 cases per 100,000 people by the summer of 2020 [61]; meanwhile, the rate of infection among migrant farmworkers, 20,015 of whom entered Ontario during the spring and summer growing seasons, was approximately 4996 cases per 100,000 people [31]. Three workers from Mexico died from the virus in Ontario during the 2020 season. As the outbreaks on southern Ontario farms continued throughout the 2020 shoulder season and into the 2021 season, Public Health Ontario [62] documented 3238 positive COVID-19 cases associated with 253 reported on-farm outbreaks by November 27, 2021 (cumulative from April 2020). Five migrant farmworkers died in 2021, shortly after arriving in Ontario, during the mandatory quarantine period [63,64]. In addition, the pandemic increased the intersecting employment strains in four ways: it amplified occupational health risks; magnified stress and tensions in employer-provided housing; increased deportability and fears of repatriation; and reduced earnings or increasing fears of income losses.

### 5.2. Transnational Employment Strain during the COVID-19 Pandemic

(i) Inadequate Protections from the Risks of Contracting the COVID-19 virus

In an effort to contain the spread of COVID-19, provinces and regional health units issued recommendations to employers in agriculture that were seeking to manage on-farm outbreaks and protect their workforce. From the workers’ perspective, these interventions can be viewed as employment resources to provide a buffer against new employment demand, that is, the requirement to work in a risky and highly contagious environment. However, this potential resource proved to be ineffective due to the virtual absence of enforcement mechanisms and the disregard for the specificities of the housing conditions among these transnational farmworkers. Ontario’s Ministry of Heath, for instance, recommended that employers limit or decrease congregate housing, organize workers into cohorts, screen workers for symptoms daily, and facilitate physical distancing, among other suggestions [65]. Quebec’s Public Health Branch of the Ministry of Health and Social Services issued even stronger recommendations and requirements, mandating that, for example, during mandatory quarantine periods upon arrival, temporary foreign workers be isolated in individual rooms with private bathrooms and be provided with means of communication and sources of entertainment (video games, radio, or television), as well as food, laundry services, and hygiene products [66]. The Public Health branch also recommended developing post-quarantine housing plans to separate contagious and potentially contagious workers from each other as well as from other migrant farmworkers, and it asked employers to avoid using dormitories with three or more beds and instead ensure workers are housed in single or double occupancy rooms [66]. 

Furthermore, regional public health units established guidelines for businesses. For instance, in 2021, the Windsor-Essex County Health Unit issued detailed requirements around physical distancing as well as the provision of personal protective equipment, cleaning products, and nutritious meals to ensure workers’ well-being during the mandatory self-isolation period; the Section 22 order (under Ontario’s *Health Protection and Promotion Act*) also detailed requirements to help limit the potential spread of COVID-19 on the worksite and in employer-provided housing after the mandatory isolation period [67]. By December 2020, Ontario’s Ministry of Labour, Training, and Skills Development conducted 375 proactive and 95 reactive COVID-19 related inspections on farms and had issued 123 COVID-19 related orders to employers in agriculture [68]. 

Despite these efforts, an inspection blitz in Southern Ontario agricultural hub Windsor-Essex in early 2021 still found one in five farms non-compliant with rules around social distancing and masking [69]. Consistent with these findings, workers reported that some of these regulations (e.g., the provision of nutritious meals, physical distancing, or the supply of personal protective equipment) were ignored on the farms where the workers we interviewed were employed. Donald told us, for instance, that on his farm, the employer originally gave each worker a box of gloves and masks for free, but subsequently, if they needed more, the cost was deducted from their pay. Some workers preferred to use a kerchief, instead of purchasing a proper mask. Donald was also concerned about the social interaction between workers from different houses. He told us that on his farm, there were four bunkhouses with eight workers in each, yet when they went shopping, they shared taxi rides with workers from other houses.

Among the workers we interviewed, ten told us that they had COVID-19 cases at work, and all but one of such cases were in Leamington. Two of the interviewed workers, both female, had contracted the disease. Rene, a worker interviewed in Niagara, Ontario told us about the spread of the virus from one region to another when his brother and his co-workers employed on a farm near St. Catharines, Ontario, were sent to work on a farm owned by the same farm owner in Leamington. As it turned out, some of the workers were already infected without knowing it. Eight days after their arrival in Leamington, some members of this group started exhibiting COVID-19 symptoms, at which point they had already spread the virus to other workers. Instead of engaging in contact tracing, the employer told the workers to conceal information about their travels. In Rene’s retelling of his brother’s story, he tells us that the supervisor advised the workers that “*if someone from the government comes and asks you where you got infected, you just tell them you don’t know*”.

Congregate housing provided by employers for their transnational labour force posed a major challenge for the workers and the public health authorities seemingly interested in containing the spread of the virus. Most workers reported that very little was done to prevent the spread of the virus in employer-provided houses. They did use disinfectants, but it was hard to distance themselves from other workers in overcrowded houses where they had to share bedrooms, bathrooms, and kitchens. Only one worker told us that their employer rented additional housing to divide up the workers to reduce overcrowding during the pandemic. While in two cases, fewer workers than usual were expected to share housing during the pandemic, this was not common. A 2021 Report of the Auditor General of Canada found that the ESDC’s 2020 inspections of farms employing migrant farmworkers showed that almost all employers were compliant with the COVID-19 regulatory requirements governing housing [70]. This highly critical report nevertheless shows that quarantine inspections had little or no evidence to support a determination of compliance, and where employers were documented to be in violation of these requirements, they were still deemed compliant [70]—an issue that only got worse in the 2021 season. Similarly, outbreak inspections were not conducted in a timely fashion, and in a majority of cases, they did not contain evidence on whether or not employers provided sick or symptomatic workers with adequate housing.

(ii) Increasing strain in employer-provided dwellings

While most employers did not take measures to ensure that the workers could maintain safe distances from each other in their houses, many took one draconian measure to try to contain the spread from the community to the workers: they forbade the employees from leaving their dwellings, that is, from socializing off-farm. Prior to the pandemic, many workers residing on farms in remote rural communities spent most of their time outside of work in their dwellings. However, the town of Leamington, where many of our interviews were conducted, is within a bicycle ride of many farms, and taxi rides, particularly when shared, are relatively inexpensive. Workers often ride their bicycles into town of Leamington enjoy sports activities, as well as culinary, social, cultural, and recreational opportunities [30]. These activities provide an emotional release from the tensions and pressure many workers experience on the farms. Once lockdown measures, such as the closure of schools as well as non-essential businesses, were introduced in the spring of 2020, however, many interviewees reported being confined to their houses. The introduction and continuation of this requirement, enforced by employers even after many COVID-19 restrictions were lifted, not only violated the workers’ rights to freedom of movement but also deprived them of the resources they needed to buffer the employment strain. Furthermore, having to spend all their free time in the company of their co-workers amplified any pre-existing tensions between them. Finally, these restrictions made the workers who found the confinement difficult to bear more precarious when employers used minor infractions as grounds for dismissal or other forms of discipline that resulted in a loss of income.

Virtually all workers interviewed in Windsor-Essex were not allowed to leave the farm during the COVID-19 pandemic, in some cases up to a year-and-a-half, despite the fact that the regional health unit (WECHU) lifted many of the original restrictions as the region progressed through the various stages of re-opening businesses, public spaces, and institutions that were ordered to stop in-person activities and service at the beginning of the pandemic. Those who were allowed to go shopping once a week were allotted a limited amount of time to purchase groceries. Some were given only 30 min, while others could spend up to one hour in the store. In most cases, the workers were transported to a store in a company van. In other reported cases, the workers had to take taxis to travel into town in groups. On one farm, only three people from the workers’ house purchased food for the rest of them. The three people in charge of shopping were rotated each week. There were also some farms on which workers were not allowed to leave at all. Instead, they filled out shopping lists, and their food was ordered for them by their employer and delivered to their doorsteps. When it was someone else purchasing food for them, they did not always get what they had selected. Some found that the food was purchased at a store where the prices were either higher than they would have preferred, or the selection of ethnic food was more limited. One worker reported that they were not allowed to step outside their house, a requirement that went beyond even the strictest lockdown measures implemented in Canada and was upheld even after some regions lifted certain restrictions.

The confinement impacted the mental health of many migrant farmworkers, who were already concerned about the health and well-being of their families left behind. Many mentioned that the workers felt stressed, depressed, restless, sad, tense, or simply bored while staying at home all the time. Some compared it to being in jail and resented that there was no opportunity for them to take their minds off of work. Ron told us that he almost went back to Mexico:

*Some of us needed a psychologist. I met with a psychologist. There was a moment when I said: “f… it”. And I locked myself up, I wasn’t going to work anymore, I stayed locked up, drinking. and I already said: “ f… it.” And in May of last year, precisely on May 10, my mother passed away, so I said: “Yes. It’s over. I’m going”.*

Ricky explains why confinement to their houses was difficult for the workers:

*We were all used to going into town when we finished work early. We would go into town. Go to a café. Or just walk, to clear my head (divagar mi mente)… And when we couldn’t go out anymore, being at the house all the time, just walking around the house on the farm property, and nothing else, not going into town, well this was a drastic change.*

Matías understood that these restrictions were meant to protect them from contracting the virus and spreading it to others. However, he also recognized that the forced confinement impacted the workers’ mental health and amplified conflicts with co-workers:

*It was very stressful. We all live in the same house and even though we don’t go out much, but the fact of knowing that we could not go out on Fridays to do shopping or on Sunday, the day to do shopping, it made us feel stressed out, feel locked up here, as if we were in prison. We were saying, why do they not let us go out as if we in prison. Why do they not let us go out? We are just here in the house and at work, and from work to the house and that’s all. It was very stressful. And it caused problems among the co-workers, and it was because we could not go out… We used to live like this before as well, but we felt even more confined during the pandemic… And so people felt very unhappy that they had to share the house with other workers, because they had to spend too much time with them without leaving, and so they felt unhappy*.

Thus, policies introduced to better protect migrant farmworkers’ health and well-being that do not account for their transnational lives can have perverse effects, amplifying the employment strain they experience. While the stay-at-home orders and mandatory isolation periods implemented during the COVID-19 pandemic sought to protect individuals and communities from the spread of the virus, they simultaneously produced additional demands (increased toll on mental health) and circumscribed integral resources (including the ability to leave their employer-provided on-farm housing during periods of rest and leisure).

(iii) Increasing migrants’ “deportability” and fears of repatriation

During the COVID-19 pandemic, many regulations and restrictions were put in place to protect citizens from this contagious virus. Ironically, some public health measures and the arbitrary ways they were enforced by the employers or migrant farmworkers had the unintended consequences of rendering these workers more precarious, thus increasing, rather than decreasing, their employment strain. For instance, workers who disobeyed the mandatory confinement rules set unilaterally by employers who forbade their workers from leaving their dwellings under any circumstances were disciplined. Matías explains: *“If someone went into town to do shopping or something, they were sent to do quarantine, and they were not paid while they were not working*.” Exceeding the recommendations made by public health authorities, such precautionary measures exemplified the power unbalances characterizing this labour importation program, which deprived workers of a voice. Ricky was one of those workers who was disciplined, as he told us:

*I left the farm one day. It was just one kilometer from the house. It was just along the road. There was nothing but farmland. I rode my bicycle. I just cycled one kilometer from the house and came back. I just needed to clear my head… But since the employer lives close to the farm … he saw me as he drove by. He stopped and asked me, “what are you doing here?” I told him that I was very bored in the house, and I needed to ride my bicycle. He asked me if I was going into town, and I said ‘no, I am not going there, boss.’”*

Still, Ricky’s employer ordered him to return to the house immediately. Even after the workers received two vaccines, his employer did not want the workers to go out to socialize with any community residents. When Ricky disobeyed, he paid a high price. While on vacation after the first year of his second two-year contract, he received a message from his employer telling him that he was not to return to work in Canada. Among the reasons for firing him, his owner mentioned that Ricky “went out with girls in town” and that in his employer’s view, this act constituted a violation of the COVID-19 restrictions.

The COVID-19 pandemic also amplified migrant farmworkers’ fears of deportation for medical reasons. Unsurprisingly, some workers in our study and their co-workers were afraid that if they were to contract the virus, they would be returned to their countries of origin. Consequently, to prevent deportation and the associated loss of income, they tried avoiding such measures as testing or monitoring, particularly if they did not experience any symptoms or if their symptoms were mild.

In the summer of 2020, when the outbreaks on farms started increasing, the Windsor-Essex County Health Unit recommended that all migrant farmworkers be tested. While this measure was adopted in an attempt to protect the agricultural labour force from the spread of the virus (and thus reduce the pandemic-induced employment strain), it ignored the unique conditions of migrant farmworkers and the insecurities of their employment. Ricky explains why many migrant farmworkers were reluctant to do so, making it evident that the possibility of losing income or even their jobs was an important consideration:

*What happened was that there were rumors that if you were to have a test, you would get the virus entering your body, and if you were to test positive, you would be sent back to Mexico. They would lock you up and then send you back to Mexico, with your contract terminated and not being able to stay in Canada. There were lots of rumors. And that’s why we were afraid to get tested, because if we tested positive, the company wouldn’t pay you, isolate you, and if you were to die, it would be very complicated, and the government wouldn’t help in any way.*

(iv) Reduced earnings and fears of income losses

Upon arrival in Canada during the pandemic, migrant farmworkers were required by the federal government to quarantine for 14 days, a public health measure motivated to curb the spread of COVID-19 among transnational workers but also to more broadly limit transmission from those travelling internationally to the domestic population. To support farmers, fish harvesters, and all food production and processing employers engaging migrant workers, the federal government announced that each employer was eligible to receive $1500 per migrant farmworker subject to self-isolation upon arrival—a subsidy to be used to cover wages or costs of accommodations during this period [71]. During their isolation period, employers of migrant farmworkers were to compensate employees for 30 h a week, at the hourly rate of pay stipulated contractually [72]. However, such interventions quickly proved insufficient in protecting migrant farmworkers during the pandemic. In terms of income support, while the mandatory and paid 14-day quarantine period upon arrival was a significant protective measure for migrant farmworkers, typically excluded from short- and long-term income supports, for workers who typically worked a 50–60 h week, a compensation equivalent to 30 h of work meant a significant loss of income and therefore remittances sent to support their families left behind. Except for the six workers we interviewed who were already in Canada when the COVID-19 pandemic started, all the other workers we interviewed had to spend two weeks in quarantine upon arrival. Furthermore, if they or their co-workers tested positive, they were placed in quarantine again. Some were placed in hotels, and some stayed in their employer’s houses or trailers. They were paid wages equal to thirty hours of work and provided food. In many cases, the cost of food that the employer provided without consulting the workers was subsequently discounted from their pay cheques.

Gisella, for instance, was frustrated to see her pay cheque being used to cover her additional living expenses when in quarantine:

*We were paid for 30 h a week during the quarantine but with this money we had to pay for the hotel, food, and swabs; there was nothing left. With that money we paid for everything.*

In Gisella’s case and that of her co-workers, her ability to support her household members was further eroded by these arbitrary measures taken by her employer.

While temporary wage-loss supports, including those provided by Quebec’s Commission des normes, de l’équité, de la santé et de la sécurité du travail (CNESST) and Ontario’s Workplace Safety and Insurance Board (WSIB), were also available to migrant farmworkers at this time, many workers in our study were not aware of these policies. They suspected that if they were to test positive for COVID-19, even if they were asymptomatic, they would be asked to quarantine, and they feared that they would not be paid any form of income support while in quarantine. Only one worker interviewed mentioned that he received WSIB coverage while in quarantine. No income support existed for migrants without legal status; one non-status migrant farmworker, her sister, and her two parents, all of whom tested positive for the virus, received no financial aid and had to rely on her uncle for survival while remaining in quarantine for two weeks. The lack of income support for migrants without legal migration status might have prevented some infected workers from abstaining from work, a necessary measure required to protect other workers. However, even for the workers on legally authorized contracts, income supports during quarantine were either inaccessible or insufficient. For the workers whose main raison d’être for participating in the Canadian TFWP was to improve the standards of living of their families, the reduced earnings (or the fear of such) during the COVID-19 pandemic was an additional source of strain that public policies did not adequately mitigate.

## 6. Conclusions

The COVID-19 pandemic revealed and exacerbated the health and economic disparities related to work for remuneration. Internationally, many migrant workers could not travel to their jobs and incurred high levels of debt due to the international travel restrictions [73], while in higher income countries like Canada, racialized workers lost their jobs at a much higher rate compared with white workers [74]. Examining those who managed to keep working during the pandemic also reveals health and economic disparities: in Canada, for instance, female workers were overrepresented in occupations with the highest risks of infections [74]. Moreoever, as this study revealed, although migrant farmworkers were exempt from travel restrictions on the basis of their work being “essential” to Canada’s food security, they were insufficiently protected from the threats posed by this global pandemic. Multiple workplace outbreaks on Canadian farms threatened the well-being of the migrant farmworkers, and some of them lost their lives when they came to work on Canadian soils. In addition, as we have argued, the “employment strains” that these workers routinely experience were amplified during the pandemic.

Building on insights from the “employment strain” perspective advanced by Lewchuk et al. [22] and Vosko [23], we enlarged this framework in our study to account for the transnational lives and relationships that the precarious-status migrant farmworkers maintain while working in Canada. Furthermore, we sought to add a structural dimension to the employment strain perspective by bringing into the frame the on-the-ground policies and measures taken by governments to mitigate employment strains (e.g., by analyzing the occupational health and safety laws, regulations, and policies for protecting abused workers) and recognizing their links to larger policies such as immigration policies such as immigration policies that grant temporary work authorization to agricultural workers, contingent upon their compliance with certain employment demands imposed on them unilaterally by employers, and separating these workers from their families.

As we have argued, pre-pandemic policies meant to provide buffers against excessive employment demands failed to account for the migrant farmworkers’ precarious legal status (i.e., their “deportability”) or their transnational living and social relations. As such, they were largely ineffective. Overall, the employment demands contributing to an unsafe work environment, including workplace harassment, long hours, pressure to increase productivity, and inadequate housing conditions, contributed to the employment strain among migrant farmworkers who had very limited employment resources available to balance the demands. One employment resource was the gratification most migrant farmworkers derive from their improved ability to support their families by remitting their wages earned in Canada that far exceed those they would earn if they stayed in their home communities. At the same time, as we have shown, the migrant farmworkers’ reliance on remittances may have compelled them to become more compliant with the harsh working and living conditions in Canada, augmenting their employment demands. Furthermore, separation from the social and emotional support of their households and communities contributed to the migrants’ employment strain, as did the requirement to share employer-provided housing that was, in many cases, less than adequate.

Applying this transnational employment strain perspective to the pandemic experiences of precarious-status migrant farmworkers revealed that the COVID-19 health crisis exacerbated already deplorable pre-pandemic working and living conditions. More specifically, the pandemic increased employment strains in four ways: by bringing forth a new risk of COVID-19 transmission in workplaces and in employer-provided dwellings; magnifying the mental and physical health risks associated with employer-provided housing; amplifying the risks of employer reprisal and repatriation; and reducing earnings and/or introducing new threats of lost income. As before the pandemic, public policy measures adopted to mitigate new risks proved to be myopic insofar as they failed to address the specific vulnerabilities confronting migrant farmworkers, particularly that of their precarious employment and legal presence in Canada and that of the living and working requirements linked to their transnational living and social relations. Thus, from a public policy perspective, in order to manage the risks posed by the ongoing pandemic and any future threats to the health security of migrant farmworkers, it is necessary to change the structural conditions that deprive them of full protections. There is, therefore, a pressing need to rethink temporary labour migration programs that tie workers to specific employers and deny them the rights reserved for legal citizens. Along with these changes, feasible yet transformative policy directions are necessary, including a more robust regime to enforce labour and health and safety rights and standards, to better support the realization of health equity among the migrant farmworkers in Canada.

## Data Availability

Access to data from this study is restricted to the research team members.

## References

[1] Caxaj S., Cohen A. (2019). “I will not leave my body here”: Migrant farmworkers’ health and safety amidst a climate of coercion. Int. J. Environ. Res. Public Health.

[2] Colindres C., Cohen A., Caxaj S. (2021). Migrant agricultural workers’ health, safety and access to protections: A descriptive survey identifying structural gaps and vulnerabilities in the interior of British Columbia, Canada. Int. J. Environ. Res. Public Health.

[3] Hennebry J., McLaughlin J., Preibisch K. (2016). Out of the loop: (In) access to health care for migrant workers in Canada. J. Int. Migr. Integr..

[4] McLaughlin J. (2009). Migration and Health: Implications for Development a Case Study of Mexican and Jamaican Migrants in Canada’s Seasonal Agricultural Workers Program.

[5] Satzewich V. (2007). Business or bureaucratic dominance in immigration policymaking in Canada: Why was Mexico included in the Caribbean seasonal agricultural workers program in 1974?. J. Int. Migr. Integr..

[6] Smith A. (2015). The bunk house rules: A materialist approach to legal consciousness in the context of migrant workers’ housing in Ontario. Osgood Hall Law J..

[7] Chartrand T., Vosko L.F. (2020). Canada’s temporary foreign worker and international mobility programs: Charting change and continuity among source countries. Int. Migr..

[8] Perry J.A. (2018). Living at work and intra-worker sociality among migrant farm workers in Canada. J. Int. Migr. Integr..

[9] Denzin N.K. (2010). Moments, mixed methods, and paradigm dialogs. Qual. Inq..

[10] Mirchandani K., Vosko L., Soni-Sinha U., Perry A., Noack A., Hall R., Gellatly M. (2018). Methodological k/nots: Designing research on the enforcement of labor standards. J. Mix. Methods Res..

[11] Hou F., Hou F., Picot G., Xu L. (2021). The Labour Market Outcomes of Economic Immigrants in the Skilled Trades. Economic and Social Reports: Statistics Canada. https://www150.statcan.gc.ca/n1/pub/36-28-0001/2021011/article/00003-eng.htm.

[12] Atkinson P., Hammersley M., Denzin N.K., Lincoln Y.S. (1994). Ethnography and participant observation. Hand-Book of Qualitative Research.

[13] Patton M. (2015). Qualitative Research and Evaluation Methods.

[14] Beausoleil C. (2020). 2014–2019: l’Immigration Temporaire au Québec. Ministère de l’Immigration, de la Francisation et de l’Intégration.

[15] Zhang Y., Ostrovsky Y., Arsenault A. (2021). Foreign Workers in the Canadian Agriculture Industry.

[16] Schreier M., Flick U. (2014). Chapter 12: Qualitative content analysis. The SAGE Handbook of Qualitative Data Analysis.

[17] Braun V., Clarke V. (2006). Using thematic analysis in psychology. Qual. Res. Psychol..

[18] Bakker A.B., Demerouti E. (2007). The job demands-resources model: State of the art. J. Manag. Psychol..

[19] Karasek R.A. (1979). Job demands, job decision latitude, and mental strain: Implications for job redesign. Adm. Sci. Q..

[20] Karasek R.A., Theorell T. (1990). Healthy Work: Stress, Productivity, and the Reconstruction of Working Life.

[21] Vosko L.F. (2010). Managing the Margins: Gender, Citizenship and the International Regulation of Precarious Employment.

[22] Lewchuk W., de Wolff A., King A., Polanyi M., Vosko L.F. (2006). The hidden costs of precarious employment: Health and the employment relationship. Precarious Employment: Understanding Labour Market Insecurity in Canada.

[23] Vosko L.F. (2006). Precarious Employment: Understanding Labour Market Insecurity in Canada.

[24] Basok T., Bélanger D., Rivas E. (2014). Reproducing deportability: Migrant agricultural workers in South-Western Ontario. J. Ethnic Migr. Stud..

[25] Vosko L.F. (2013). National sovereignty and transnational labour: The case of Mexican seasonal agricultural workers in British Columbia, Canada. Ind. Relat. J..

[26] Vosko L.F. (2019). Disrupting Deportability: Transnational Workers Organize.

[27] Binford L. (2009). From fields of power to fields of sweat: The dual process of constructing temporary migrant labour in Mexico and Canada. Third World Q..

[28] Orkin A.M., Lay M., McLaughlin J., Schwandt M., Cole D. (2014). Medical repatriation of migrant farm workers in Ontario: A descriptive analysis. CMAJ Open.

[29] Hennebry J.L., Williams G. (2015). Making vulnerability visible: Medical repatriation and Canada’s migrant agricultural workers. CMAJ.

[30] Basok T., George G. (2020). “We are part of this place, but I do not think I belong”. Temporariness, social inclusion and belonging among migrant farmworkers in Southwestern Ontario. Int. Migr..

[31] Vosko L.F., Spring C. (2021). COVID-19 outbreaks in Canada and the crisis of migrant farmworkers’ social reproduc-tion: Transnational labour and the need for greater accountability among receiving states. J. Int. Migr. Integr..

[32] Tsui E.K., LaMonica M., Hyder M., Landsbergis P., Zelnick J., Baron S. (2021). Expanding the conceptualization of support in low-wage carework: The case of home care aides and client death. Int. J. Environ. Res. Public Health.

[33] Goldring L., Landolt P. (2021). From illegalised migrant toward permanent resident: Assembling precarious legal status trajectories and differential inclusion in Canada. J. Ethnic Migr. Stud..

[34] Gatehouse J. (2020). How Undocumented Migrant Workers Are Slipping through Ontario’s COVID-19 Net. CBC.

[35] IRCC (2022). Canada-Work Permit Holders by Program, Country of Citizenship and Year in which Permit(s) Became Effective, 2002–2021.

[36] Rajkumar D., Berkowitz L., Vosko L.F., Preston V., Latham R. (2012). At the temporary permanent divide: How Canada produces temporariness and makes citizens through its security, work, and settlement policies. Citizensh. Stud..

[37] McLaughlin J. (2010). Classifying the “ideal migrant worker”: Mexican and Jamaican transnational farmworkers in Canada. J. Glob. Hist. Anthropol..

[38] McLaughlin J., Wells D., Mendiburo A.D., Lyn A., Vasilevska B. (2017). ‘Temporary workers’, temporary fathers: Transnational family impacts of Canada’s Seasonal Agricultural Worker Program. Ind. Relat..

[39] Wells D., McLaughlin J., Lyn A., Mendiburo A.D. (2014). Sustaining Precarious Transnational Families: The Significance of Remittances from Canada’s Seasonal Agricultural Workers Program. Just Labour.

[40] ESDC (2021). Contract for the Employment in Canada of Seasonal Agricultural Workers from Mexico.

[41] Canadian Agricultural Injury Reporting (CAIR) (2016). Canadian Agricultural Injury Reporting: Agriculture-Related Fatalities in Canada.

[42] McLaughlin J., Hennebry J. (2011). Backgrounder on Health and Safety for Migrant Farmworkers.

[43] Champagne S.R. (2022). Un Premier Travailleur Agricole Mexicain Fait Reconnaître Son Cancer Lié Aux Pesticides.

[44] McLaughlin J., Tew M., Huesca E., Premji S. (2018). Compounded vulnerabilities and creative strategies: Occupational health of temporary foreign agricultural workers. Sick and Tired: Health and Safety Inequalities.

[45] Gravel S., Villanueva F., Bernstein S., Hanley J., Villarreal D.C., Ostiguy E. (2014). Les mesures de santé et sécurité au travail auprès des travailleurs étrangers temporaires: Le cas du secteur agro-alimentaire. Perspect. Interdiscip. Trav. Santé.

[46] Hanley J., Gravel S., Lippel K., Koo J.H. (2014). Pathways to healthcare for migrant workers: How can health entitle-ment influence occupational health trajectories?. Perspect. Interdiscip. Trav. Santé.

[47] McLaughlin J., Hennebry J., Haines T. (2014). Paper versus practice: Occupational health and safety protections and realities for temporary foreign agricultural workers in Ontario. Perspect. Interdiscip. Trav. Santé.

[48] Cedillo L., Lippel K., Nakache D. (2019). Factors influencing the health and safety of temporary foreign workers in skilled and low-skilled occupations in Canada. New Solut. J. Environ. Occup. Health Policy.

[49] Basok T. (2002). Tortillas and Tomatoes: Transmigrant Mexican Harvesters in Canada.

[50] Carvajal Gutierrez L., Johnson T.G. (2016). The impact of remittances from Canada’s seasonal workers programme on Mexican farms. Int. Labour Rev..

[51] IRCC (2020). Open Work Permits for Vulnerable Workers [R207.1-A72]—International Mobility Program. https://www.canada.ca/en/immigration-refugees-citizenship/corporate/publications-manuals/operational-bulletins-manuals/temporary-residents/foreign-workers/vulnerable-workers.html.

[52] Vosko L.F., Tucker E., Casey R. (2019). Enforcing employment standards for migrant agricultural workers in Ontario, Canada: Exposing underexplored layers of vulnerability. Int. J. Comp. Labour Law Ind. Relat..

[53] Preibisch K., Hennebry J. (2011). Temporary migration, chronic effects: The health of international migrant workers in Canada: Table 1. Can. Med. Assoc. J..

[54] Díaz Mendiburo A., McLaughlin J. (2016). Structural vulnerability and health among seasonal agricultural workers in Canada. Alterities.

[55] Basok T. (2004). Post-national citizenship, social exclusion and migrants rights: Mexican seasonal workers in Canada. Citizsh. Stud..

[56] Hennebry J.L. (2012). Permanently Temporary? Agricultural Migrant Workers and Their Integration in Canada.

[57] Aziz A. (2020). A Promise of Protection? An Assessment of IRCC Decision-Making under the Vulnerable Worker Open Work Permit Program.

[58] Marsden S., Tucker E., Vosko L.F. (2021). Flawed by design? A case study of federal enforcement of migrant workers’ labour rights in Canada. Can. Labour Employ. Law J..

[59] Mojtehedzadeh S. (2020). Migrant Workers with COVID-19 Must not be Allowed to Work, Health Experts Say—As Infected Worker Count Surpasses 1000. Toronto Star.

[60] Baum K., Grant T. (2020). Ottawa Didn’t Enforce Rules for Employers of Migrant Workers during Pandemic. The Globe and Mail. https://www.theglobeandmail.com/canada/article-how-ottawas-enforcement-regime-failed-migrant-workers-during-the/.

[61] Detsky A.S., Bogoch I.I. (2020). COVID-19 in Canada: Experience and response. JAMA.

[62] Public Health Ontario (PHO) (2021). COVID-19 in Ontario: Focus on November 21, 2021 to November 27, 2021. https://files.ontario.ca/moh-covid-19-weekly-epi-report-en-2021-11-27.pdf.

[63] Caxaj S., Tran M., Tew M., Mayell S., McLaughlin J., Rawal S., Cole D., Vosko L.F. (2022). Key Findings and Recommendations from a Study of Coroner’s Files of Migrant Agricultural Workers’ Deaths in Ontario from January 2020 to June 2021. https://www.ohcow.on.ca/wp-content/uploads/2022/02/coroner_study_key_findings_recs_final_v2.pdf.

[64] Migrant Workers Alliance for Change (MWAC) (2021). Migrant Farm Worker Dies of COVID in Government Mandated Quarantine. https://migrantworkersalliance.org/press/5thfarmworkerdeathmay2021/.

[65] Ministry of Health (2020). COVID-19 Guidance: Workplace and Living Settings for Seasonal International Agriculture Workers (IAWs). https://health.gov.on.ca/en/pro/programs/publichealth/coronavirus/docs/COVID-19_Farm_Outbreak_guidance.pdf.

[66] Institut National de Santé Publique du Québec (INSPQ) (2021). Aide-Mémoire 2021 Pour l’Arrivée au Québec de Travailleurs Étrangers Temporaires du Secteur Bioalimentaire. https://www.cisss-bsl.gouv.qc.ca/sites/default/files/fichier/2021-04-16_aide-memoire_travailleurs_etrangers_temporaires_fr_2021.pdf.

[67] Windsor-Essex County Health Unit (WECHU) (2020). Class Order Made Pursuant to Section 22(5.0.1) of the Health Protection and Promotion Act, R.S.O. 1990, c.H.7. https://www.wechu.org/sites/default/files/edit-resource/em-section-22-class-action-order-farms/class-action-order-farms-apr-2021.pdf.

[68] Government of Ontario (2021). Data Request G-2021-00016 (COVID Related Request), Index #DR21-074.

[69] Martin M. (2021). One in Five Farms Not Compliant with COVID Rules, Early Inspections Find.

[70] Auditor General of Canada (2021). Report 13: Health and Safety of Agricultural Temporary Foreign Workers in Canada during the COVID-19 Pandemic. https://www.oag-bvg.gc.ca/internet/docs/parl_oag_202112_02_e.pdf.

[71] Agriculture and Agri-Food Canada (AAFC) (2020). Keeping Canadians and Workers in the Food Supply Chain Safe. https://www.canada.ca/en/agriculture-agri-food/news/2020/04/keeping-canadians-and-workers-in-the-food-supply-chain-safe.html.

[72] ESDC (2021). Hire a Temporary Foreign Agricultural Worker.

[73] Jones K., Mudaliar S., Piper N. (2021). Locked Down and in Limbo: The Global Impact of COVID-19 on Migrant Worker Rights and Recruitment. ILO Report. https://www.ilo.org/wcmsp5/groups/public/---ed_protect/---protrav/---migrant/documents/publication/wcms_821985.pdf.

[74] Alook A., Block S., Galabuz G.E. (2021). A Disproportionate Burden COVID-19 Labour Market Impacts on Indigenous and Racialized Workers in Canada.

